# Comparative Use of a Caribbean Mesophotic Coral Ecosystem and Association with Fish Spawning Aggregations by Three Species of Shark

**DOI:** 10.1371/journal.pone.0151221

**Published:** 2016-05-04

**Authors:** Alexandria E. Pickard, Jeremy J. Vaudo, Bradley M. Wetherbee, Richard S. Nemeth, Jeremiah B. Blondeau, Elizabeth A. Kadison, Mahmood S. Shivji

**Affiliations:** 1 Guy Harvey Research Institute, Guy Harvey Oceanographic Center, Nova Southeastern University, 8000 N Ocean Drive, Dania Beach, FL, 33004, United States of America; 2 Department of Biological Sciences, University of Rhode Island, Kingston, RI, 02881, United States of America; 3 Center for Marine and Environmental Studies, University of the Virgin Islands, #2 John Brewers Bay, St. Thomas, USVI, 00802, United States of America; University of California Santa Cruz, UNITED STATES

## Abstract

Understanding of species interactions within mesophotic coral ecosystems (MCEs; ~ 30–150 m) lags well behind that for shallow coral reefs. MCEs are often sites of fish spawning aggregations (FSAs) for a variety of species, including many groupers. Such reproductive fish aggregations represent temporal concentrations of potential prey that may be drivers of habitat use by predatory species, including sharks. We investigated movements of three species of sharks within a MCE and in relation to FSAs located on the shelf edge south of St. Thomas, United States Virgin Islands. Movements of 17 tiger (*Galeocerdo cuvier*), seven lemon (*Negaprion brevirostris*), and six Caribbean reef (*Carcharhinus perezi*) sharks tagged with acoustic transmitters were monitored within the MCE using an array of acoustic receivers spanning an area of 1,060 km^2^ over a five year period. Receivers were concentrated around prominent grouper FSAs to monitor movements of sharks in relation to these temporally transient aggregations. Over 130,000 detections of telemetered sharks were recorded, with four sharks tracked in excess of 3 years. All three shark species were present within the MCE over long periods of time and detected frequently at FSAs, but patterns of MCE use and orientation towards FSAs varied both spatially and temporally among species. Lemon sharks moved over a large expanse of the MCE, but concentrated their activities around FSAs during grouper spawning and were present within the MCE significantly more during grouper spawning season. Caribbean reef sharks were present within a restricted portion of the MCE for prolonged periods of time, but were also absent for long periods. Tiger sharks were detected throughout the extent of the acoustic array, with the MCE representing only portion of their habitat use, although a high degree of individual variation was observed. Our findings indicate that although patterns of use varied, all three species of sharks repeatedly utilized the MCE and as upper trophic level predators they are likely involved in a range of interactions with other members of MCEs.

## Introduction

Mesophotic coral reefs (MCEs), loosely defined as ranging in depth from 30 m to depths where light levels are too low for zooxanthellae to sustain coral growth via photosynthesis [1], constitute a considerable amount of reef habitat globally. Although mapping of MCEs is in its infancy, over 186,000 km^2^ of potential MCE habitat has been identified in U.S. waters alone [2]. However, as a result of technological challenges and limitations of safely working at depth, studies on MCE ecology have lagged greatly behind those for shallow coral reefs [3–6].

Large sharks and upper level predators [7] likely play an important role in structuring marine ecosystems [8,9,10], including coral reef systems [11,12]. Because movement patterns delineate the spatial and temporal extent of species interactions, understanding the movements and habitat use of sharks is essential for assessing their role in coral reef ecosystems. Although shark movements and habitat use have been studied in shallow coral reef systems [13–17], little is known about their use of MCEs [6]. Given the large amount of MCE habitat that exists, their potential importance as refuges and sources of recruits for shallow water reef species, and concerns about declining shark populations globally [18,19], studies of shark utilization of MCEs are of increasing interest and importance in reference to both shark populations and MCE ecology.

Many factors potentially influence movements and habitat use of MCEs by sharks. Abundance and distribution of prey may vary both spatially and temporally and have been shown to dramatically influence movements of predators [13,14]. Fish spawning aggregations (FSAs), composed of hundreds to thousands of individuals occur in MCEs on highly predictable temporal and spatial scales [20] and therefore may represent intense predator-prey interactions that strongly influence both predator and prey populations. Patterns of use of MCEs by large predators also relate to competitive and symbiotic relationships among reef inhabitants, and are indicators of levels of nutrient transport and connectivity among marine ecosystems [6,21,22].

The insular slope reefs of Puerto Rico and the United States Virgin Islands (USVI) are predominantly MCEs [23] and are also characterized by an abundance of sharks [24]. These ecosystems are relatively undisturbed by anthropogenic influences because of their considerable distance from land, runoff and other pollutants [23,25]. In addition, several well-studied FSAs are located in the MCE south of St. Thomas, USVI [20,24]. This ecosystem presents a good opportunity to examine movements of sharks within a MCE and in relation to FSAs. Here we utilize passive acoustic telemetry to investigate overall use of the MCE and orientation towards FSAs by three species of highly mobile, large sharks (lemon, *Negaprion brevirostris*; tiger, *Galeocerdo cuvier*; and Caribbean reef, *Carcharhinus perezi*) on the Puerto Rico-Virgin Islands platform. Our goals were to: 1) quantify spatio-temporal patterns of MCE reef use for each species of shark, and 2) examine temporal orientation of sharks towards grouper FSAs off St. Thomas, USVI.

## Methods

### Study Site

**Fig 1:**
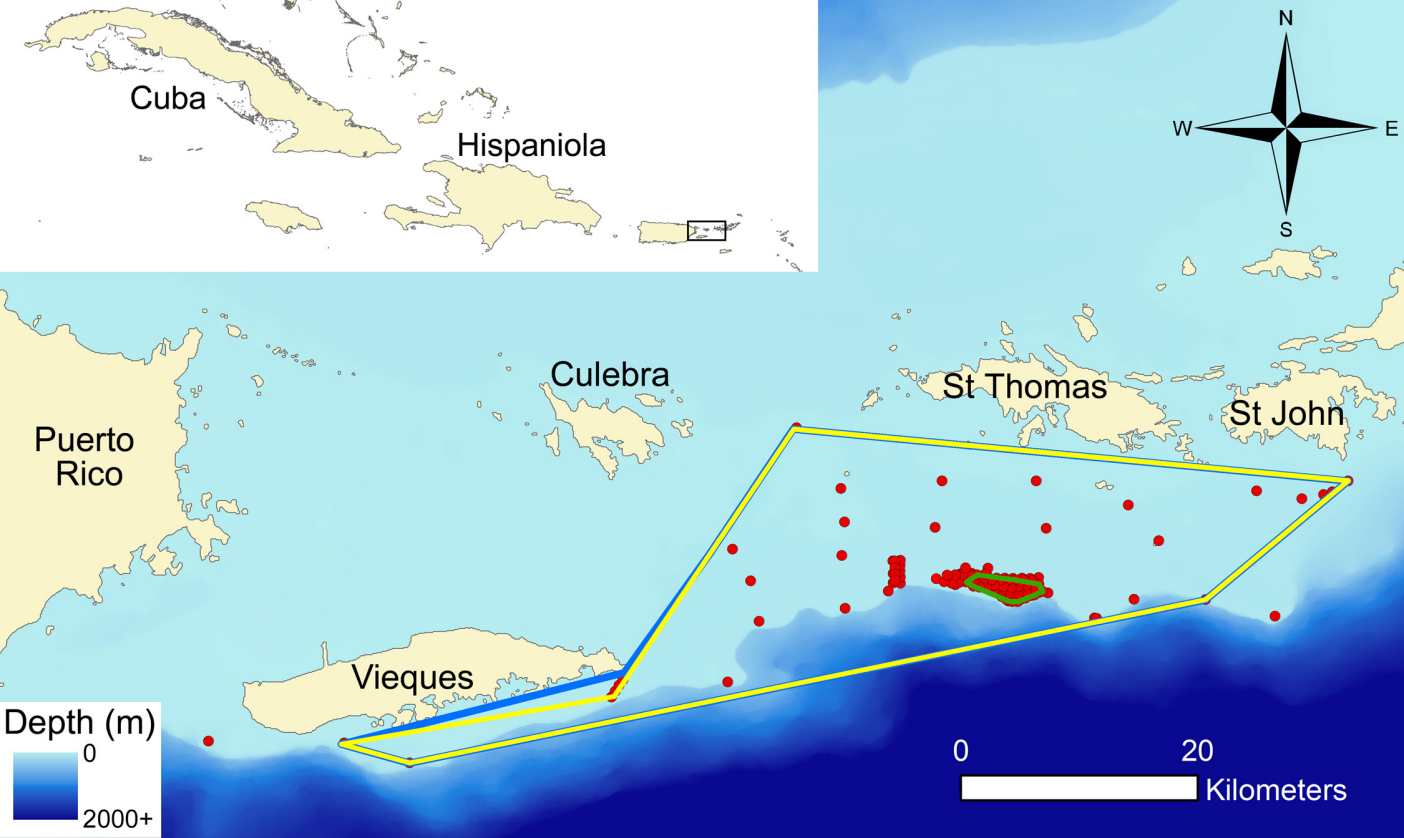
Acoustic array located along the southern edge of the Puerto Rico-Virgin Islands platform. Red circles represent locations of acoustic receivers. Minimum convex polygons (MCP) for three species of sharks quantified by acoustic tracking are indicated for all tiger sharks (blue line, 887 km^2^), lemon sharks (yellow line, 863 km^2^) and Caribbean reef sharks (green line, 8 km^2^).

This study was conducted within the MCE located along the southern insular shelf edge of the Puerto Rico-Virgin Islands platform, spanning ~100 km from St. John, USVI, to Vieques, Puerto Rico and adjacent to the 4000 m deep Virgin Islands Trough (Fig 1). This well-developed linear reef is located at depths of 30–45 m and is topographically complex, consisting of large (1–2 m) coral ridge colonies of *Montastraea* spp. [23,25].

**Fig 2:**
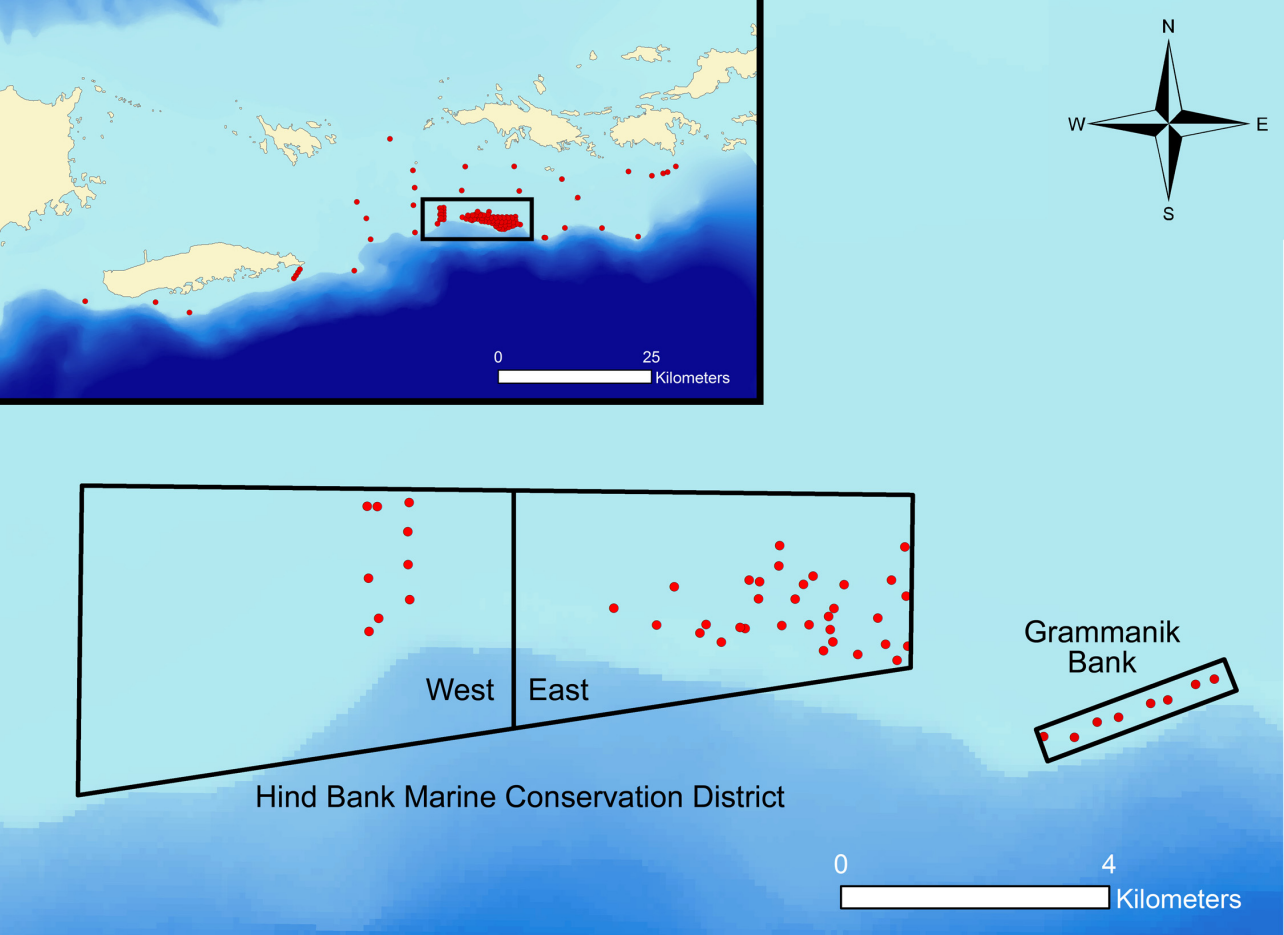
Acoustic receiver locations within two FSA sites south of St. Thomas, USVI. Boundaries of the Hind Bank Marine Conservation District (MCD) and the Grammanik Bank fishing area closure are shown. Receivers were aggregated within the Hind Bank MCD along a general boundary of MCD East and MCD West. Each red circle represents the location of a receiver

The MCE includes two fishing closure areas 12 km south of St. Thomas: the Hind Bank Marine Conservation District (MCD) and the Grammanik Bank (Fig 2). Hind Bank MCD (18°12N, 65°00W), established in December 1999 to protect a red hind spawning aggregation site, is a 44.6 km^2^ area permanently closed to fishing [26] and encompasses 23.6 km^2^ of dense coral [27]. The Hind Bank MCD is used by red hind grouper (*Epinephelus guttatus*) for spawning from December to February [26]. Grammanik Bank (18°11N, 64°57W), established in 2005, encompasses 1.5 km^2^ of MCE and is closed to fishing between 1 February and 30 April, which coincides with yellowfin (*Mycteroperca venenosa*) and Nassau (*Epinephelus striatus*) grouper spawning season but also hosts other aggregating species including yellowmouth (*Mycteroperca interstitialis*), and tiger grouper (*M*. *tigris*), cubera (*Lutjanus cyanopterus*) and dog (*L*. *jocu*) snapper and Bermuda chub (*Kyphosis sectatrix*) [24,28,29]. El Seco, a third FSA site located southeast of Vieques Island, is a multi-species aggregation for tiger grouper (February to May) and possibly yellowfin grouper [30] as well as cubera and dog snapper and white margate (*Haemulon album*) (R. Nemeth personal observation).

### Sampling

A total of 30 sharks of three species: 17 tiger, seven lemon, and six Caribbean reef, were caught on bottom longlines (soak times: 3–4 hours) set adjacent to the Hind Bank MCD and Grammanik Bank FSAs. Longlines were 366 m long with 25 360-cm long gangions (180 cm of 6-mm polypropylene rope and 180 cm of 3-mm stainless steel cable) terminating in a 16/0 recurved hook. Sharks were brought on board the fishing vessel using a customized slide that allowed easy retrieval and release of sharks with minimal handling. A seawater hose was placed in the mouth of sharks to irrigate the gills while sharks were measured (length refers to total length), sexed, and tagged with a conventional ID dart tag.

Acoustic transmitters, V16-3H, (16 x 64 mm, Vemco Ltd., Nova Scotia) were used for sharks >150 cm and V13-1H (13 x 36 mm) for sharks <150 cm. Transmitters emitted unique coded acoustic signals at 69 kHz with delays of 40–120 seconds and had an estimated battery life of 550–2350 days. Transmitters coated with a 50:50 mixture of paraffin:beeswax to smooth edges and to make the transmitters inert, were surgically implanted into the body cavity [31]. The surgical incision was closed with 2–3 nylon sutures and the wound was doused with betadine. Surgeries were performed by BW and MS, both of whom had completed the Collaborative Institutional Training Initiative course for work on vertebrates. No complications occurred during surgery. Anesthetic was not used because of the large size of the sharks, because surgery was performed out of the water and to allow rapid recovery of sharks when returned to the water upon release. The entire procedure from bringing the shark on board to release was completed in 10 minutes or less. All sharks appeared healthy and swam away upon release.

### Acoustic Monitoring

An array of acoustic receivers (VR2W, Vemco) that included 127 different locations was used to monitor shark and grouper movements around FSAs (78 locations) and at distant locations within the MCE between 2007 and 2011, encompassing an area of approximately 1,060 km^2^ (Fig 1). Receivers were anchored to the substrate with a cement block and suspended 15 m off the bottom on polypropylene line buoyed by a plastic float. When receivers detected a transmitter, detection time, date, and transmitter ID were recorded and archived. Receivers were periodically retrieved and archived data were downloaded. The array was initially deployed to monitor fine-scale movement of grouper during the spawning season, resulting in high receiver density near FSAs during spawning, followed by relocation of redundant FSA receivers to additional MCE habitat during the non-spawning season.

### Analysis

#### MCE Use

To quantify size of the MCE habitat used by individual sharks, minimum convex polygons (MCP), modified to exclude land (Vieques Island, Puerto Rico) were constructed for each shark as determined by location of all acoustic receivers on which an individual was detected during the study (ArcGIS 10.0 Spatial Analyst tool, Minimum Bounding Geometry; Convex Hull). The relationship between shark size and size of MCP and also the relationship between track duration (i.e. number of days tracked) and size of MCP were examined for tiger and lemon sharks using linear regression. Limited sample sizes for Caribbean reef sharks did not allow examination of these relationships. The extent of detections from all individuals of a given species was used to construct MCPs for each of the three species of shark.

To further characterize use of the MCE we quantified three features of the detection history for each individual: 1) “track duration” (span of days between first and last detections on receivers within the entire array); 2) temporal pattern of “presence” (number of consecutive days detected on at least one receiver within the array) and 3) temporal pattern of “absence” (number of consecutive days not detected within the array). Patterns of presence and absence together constituted discrete alternating intervals of presence (detected x number of days in a row) and absence (no detections over a span of x number of days in a row) exhibited by each individual. A sequence of consecutive days present within the array was considered a unique “presence value” and each sequence of consecutive days not detected was treated as a unique “absence value”, both measured in days. To examine patterns of presence/absence histograms depicting frequency of presence values and of absence values were constructed for each individual. Additionally, average presence values and absence values were calculated for individuals and these values combined to obtain an average presence value and an average absence value for each of the three species as a whole. Average presence values and absence values were compared among species using a one-way ANOVA and Tukey’s post-hoc test. To determine if absence values conformed to a random distribution, the frequencies of absence values were compared to a Poisson distribution using a chi square test.

### Association with the FSA

Because of the proximity of receivers at FSAs within the Hind Bank MCD and Grammanik Bank and the mobility of telemetered sharks, individuals were frequently detected on multiple receivers at FSAs on the same day, often within a short time. Therefore, for purposes of monitoring presence of sharks at FSAs, the Hind Bank MCD and Grammanik Bank FSAs were considered a single FSA and data from receivers in proximity to both FSAs was combined for analyses directed at association of sharks with FSAs. The combined Hind Bank/Grammanik Bank spawning sites are hereafter referred to as the FSA.

The relationship between occurrence of sharks at spawning sites and the grouper spawning activity was examined by comparing occurrence of sharks at the FSA on a monthly basis during grouper spawning months (December—May) and non-spawning months (June—November). To quantify occurrence of sharks at the FSA on a monthly basis, a “residence index” was calculated for each individual shark for each month tracked [32]. Residence index was calculated as the number of days during a month that a shark was detected at the FSA as a proportion of the total number of days in that month. The proportional residence indices standardized values to account for differences in the number of days within months and allowed comparison of residence indices among all months. An average monthly residence index for spawning season was calculated by combining residence indices from all individuals during all months when spawning occurred over the span of the five year study. An average monthly residence index for non-spawning was calculated in a similar manner based on data from non-spawning months. Average spawning and non-spawning residence indices were compared for each species with a t-test.

Activity near FSAs was also examined by calculating presence and absence values at the FSA site using the same criteria for calculation of presence and absence used for the entire MCE (i.e. presence = detected x number of days in a row; absence = no detections over a span of x number of days in a row). Mean presence and absence values at the FSA during grouper spawning were compared with mean values for non-spawning season for each shark using a paired t-test.

To determine if sharks disproportionately visited any of the three distinct spawning sites (Hind Bank MCD, Grammanik Bank and El Seco), the percentage of days a given species was detected during the spawning season was calculated for each receiver in each of the three spawning areas by dividing the number of days sharks were detected on a receiver by the total number of days that the receiver was listening during the spawning season. The percentages were arcsine transformed and compared among the three spawning areas using a one-way ANOVA. All statistical analyses were evaluated at a significance level of 0.05.

This study was conducted with approval from the US Virgin Islands Department of Planning and Natural Resources Division of Fish and Wildlife scientific collection permit (STT-011-07) and National Marine Fisheries Service (SHK-LOA-07-05). At the time this field work (animal handling) was performed, the University of the Virgin Islands did not have an Animal Welfare Committee and an institutional permit was not required. Rather, all animal handling procedures were conducted using guidelines established by the American Fisheries Society and American Society of Ichthyology and Herpetology, and all efforts were made to minimize animal stress and suffering.

## Results

The 30 sharks (17 tiger, seven lemon, six Caribbean reef) implanted with transmitters generated over 130,000 unique detections during the five years of the study. Eighteen sharks (10 tiger, six lemon, two Caribbean reef) were detected a sufficient number of times to provide data suitable to characterize and compare their movements, with 10 individuals monitored more than two years and six individuals with tracks approaching or exceeding three years (Table 1). The sharks used for analyses were detected over periods of 91–1,339 d, with a mean of 703 ± 450 d (± SD) detected. The remaining 12 sharks were detected on receivers for less than three months or not at all and were not included in analyses. All sharks carrying transmitters, but not included in analyses were detected on multiple receivers following their release with the exception of two tiger sharks, two Caribbean reef sharks and one lemon shark that were never detected. Survival of the lemon and Caribbean reef sharks is unknown, but both of the tiger sharks were also fitted with satellite transmitters and were both detected by satellites at locations in excess of 75 km from the acoustic array, indicating survival of these individuals.

**Table 1:** Sharks tagged with acoustic transmitters, number of detections on acoustic receivers comprising the mesophotic reef receiver array and track details.

Species	Sex	TL (cm)	Detections	Track Duration (days)	MCP Area (km^2^)
Lemon	F	275	28070	847	219.3
Lemon	F	245	3348	1339	163.4
Lemon	F	278	753	110	341.2
Lemon	F	266	1421	1149	149.2
Lemon	M	251	916	160	37.1
Lemon	F	249	1530	521	5.6
Tiger	F	316	1792	264	2.3
Tiger	F	298	4330	850	387.5
Tiger	F	315	965	878	219.3
Tiger	F	275	151	551	52.7
Tiger	M	270	4842	1070	324.4
Tiger	M	330	431	1068	185.2
Tiger	F	260	407	179	250.5
Tiger	M	347	4056	1186	290.9
Tiger	F	278	124	166	97.8
Tiger	M	341	15	91	35.8
C. reef	M	157	17459	1336	5.4
C. reef	M	132	63385	899	5.5

MCP = Minimum convex polygon

C. reef = Caribbean reef.

### MCE Use

Use of the MCE varied considerably among both individuals and species on spatial and temporal scales and in terms of association with FSAs. For both lemon and tiger sharks, some individuals were detected on receivers throughout nearly the entire acoustic array, with MCPs nearly as large as the complete array, but other individuals were detected primarily on receivers near the FSA, with much smaller MCPs (Table 1). One lemon and one tiger shark were also detected on nearshore acoustic receivers deployed by researchers monitoring movements of reef fishes in several bays off the island of St. John, also indicating extensive movements of both species throughout the MCE of the Puerto Rico-Virgin Islands platform. Size of individual MCPs therefore varied over a wide range for both lemon (5.6–341.2 km^2^) and tiger sharks (20.3–387.5 km^2^). There was a large amount of overlap of activity space among the three species and among individual sharks with no evidence of partitioning of space within the MCE.

The two Caribbean reef sharks were both detected on most of the same receivers, resulting in small MCPs of similar size (5.4–5.5 km^2^) and location, both near their tagging sites and the FSA. Although average MCP size for tiger sharks (186.4 ± 129.8 km^2^) was larger than that of lemon sharks (152.69 ± 122.52 km^2^), the difference was not significant (t-test, *p* = 0.98). Linear regression revealed a significant positive relationship between track duration and size of MCPs for tiger sharks (*p* = 0.03), but not for lemon sharks (*p* = 0.96). The relationship between shark size and MCP size was not significant for either tiger (p = 0.69) or lemon sharks (*p* = 0.06). At the species level, both tiger and lemon sharks ranged over nearly the entire area of MCE monitored by the acoustic array (MCP areas of 887 km^2^ and 863 km^2^, respectively), whereas Caribbean reef sharks were detected over a substantially smaller area (roughly 8 km^2^) around the Grammanik Bank FSA (Fig 1).

The three species of sharks also exhibited variable temporal patterns of MCE use. All three species demonstrated extensive use of the MCE either spatially or temporally as indicated by the prolonged duration of tracks (Table 1) and extent of receivers upon which they were detected (Fig 1). Three of the six lemon sharks were detected on receivers for greater than two years and four of six were tracked for more than a year, resulting in an average track duration of 1.9 years (688 ± 510 d). Five of 10 tiger sharks used in analyses were detected greater than two years, with average track duration approaching two years (630 ± 429 d). The two Caribbean reef sharks were both detected for long periods of time; 2.5 and 3.7 years, respectively (average track duration of 3.1 years).

**Fig 3:**
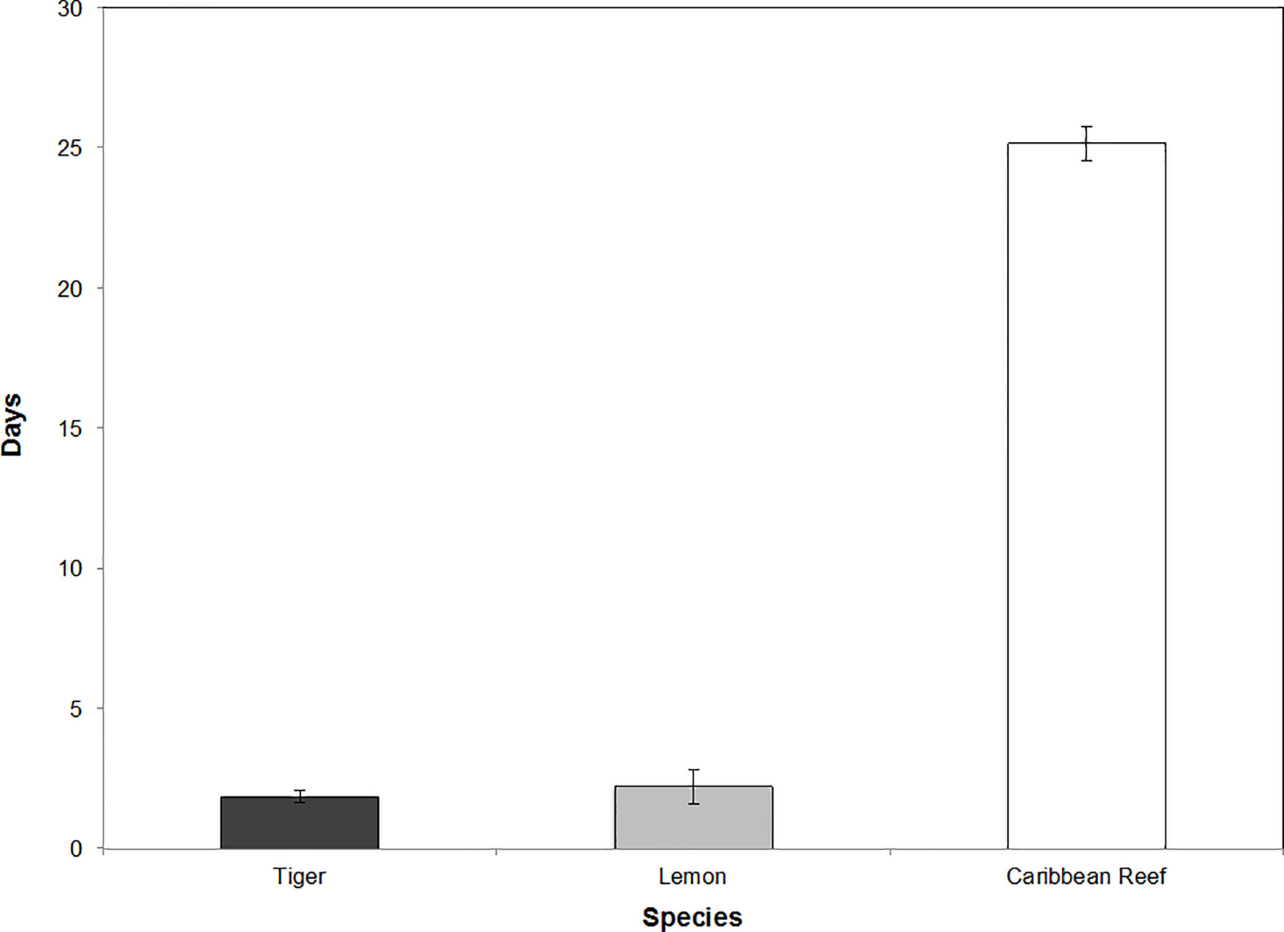
Average presence values for three species of shark within MCE habitat monitored by the acoustic array. Presence values (days) = average number of consecutive days detected on any receiver within the acoustic array for tiger (1.8), lemon (2.2), and Caribbean reef sharks (25.2). Bars represent means ± standard error.

For 17 of the 18 sharks analyzed, the distribution of the number of days between successive days detected within the MCE array differed from a random distribution (all *p* < 0.01), indicating that arrival and departure of sharks from the MCE was not random. Presence values (number of consecutive days detected on receivers) for sharks within the MCE was highly variable among individuals, and also varied among species. Average presence value for Caribbean reef sharks (25.2 ± 0.6 d) was significantly greater than those for lemon (2.2 ± 0.6 d) and tiger sharks (1.8 ± 0.2 d) (Fig 3) (*p* < 0.0001), however average presence values of lemon and tiger sharks did not differ significantly (*p* = 0.67). Although presence values of lemon and tiger sharks were low, the pattern of MCE use for all individuals of both species was characterized by brief periods of presence on the reef followed by slightly longer periods without detections, when they may have been absent from the reef.

**Fig 4:**
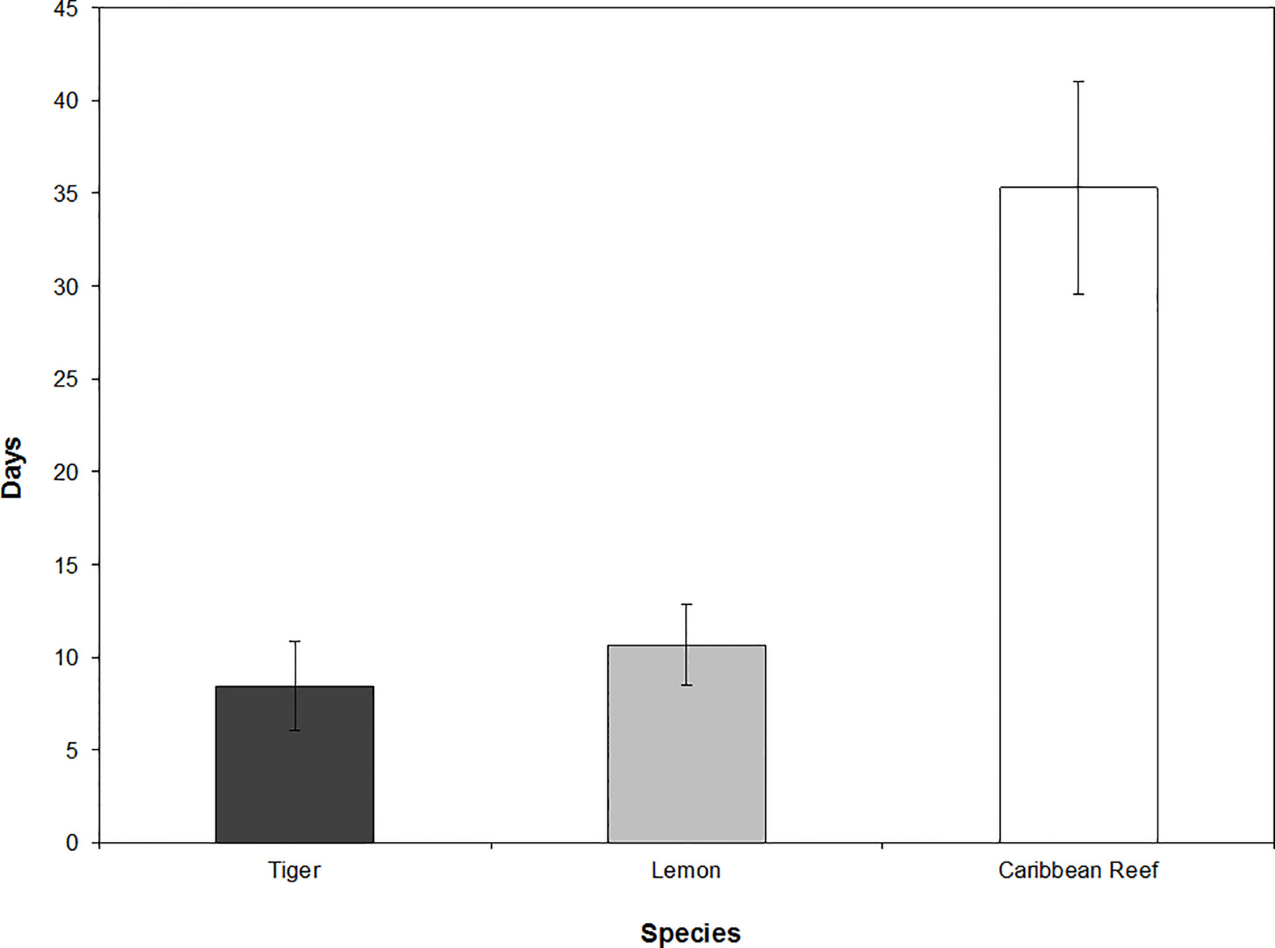
Average absence values for three species of sharks within MCE habitat monitored by the acoustic array. Absence value = average number of consecutive days not detected within the acoustic array for tiger (8.4 days), lemon (10.6 days), and Caribbean reef sharks (35.3 days). Bars represent means ± standard error.

Absence values (number of consecutive days not detected) also varied among species, with the average absence time of Caribbean reef sharks (35.3 ± 5.7 d) significantly greater than those of lemon (10.6 ± 2.2 d) and tiger sharks (8.4 ± 2.4 d) (Fig 4) (*p* = 0.001). There was no significant difference between average absence times for lemon and tiger sharks (*p* = 0.507). Presence and absence values combined indicated that Caribbean reef sharks were both present and absent for substantially longer periods of consecutive days than either lemon or tiger sharks.

Dissimilarities in use of the MCE among the three species of sharks also resulted in large differences in the number of days between consecutive detections. A very high proportion of detections for Caribbean reef sharks (96.1 ± 0.40%) occurred on consecutive days (i.e. no gaps between days detected), whereas only 52.8 ± 16.2% of detections occurred on consecutive days for lemon sharks and 44.8 ± 17.7% for tiger sharks. There were also major differences in the number of days that sharks were absent between detections, as indicated by the much higher proportion of absence values that fell between 1–7 d for tiger (42.1 ± 26.7%) and lemon (30.9 ± 24.3%) sharks in comparison to Caribbean reef sharks (2.6 ± 2.5%).

### Association with the FSA

**Fig 5:**
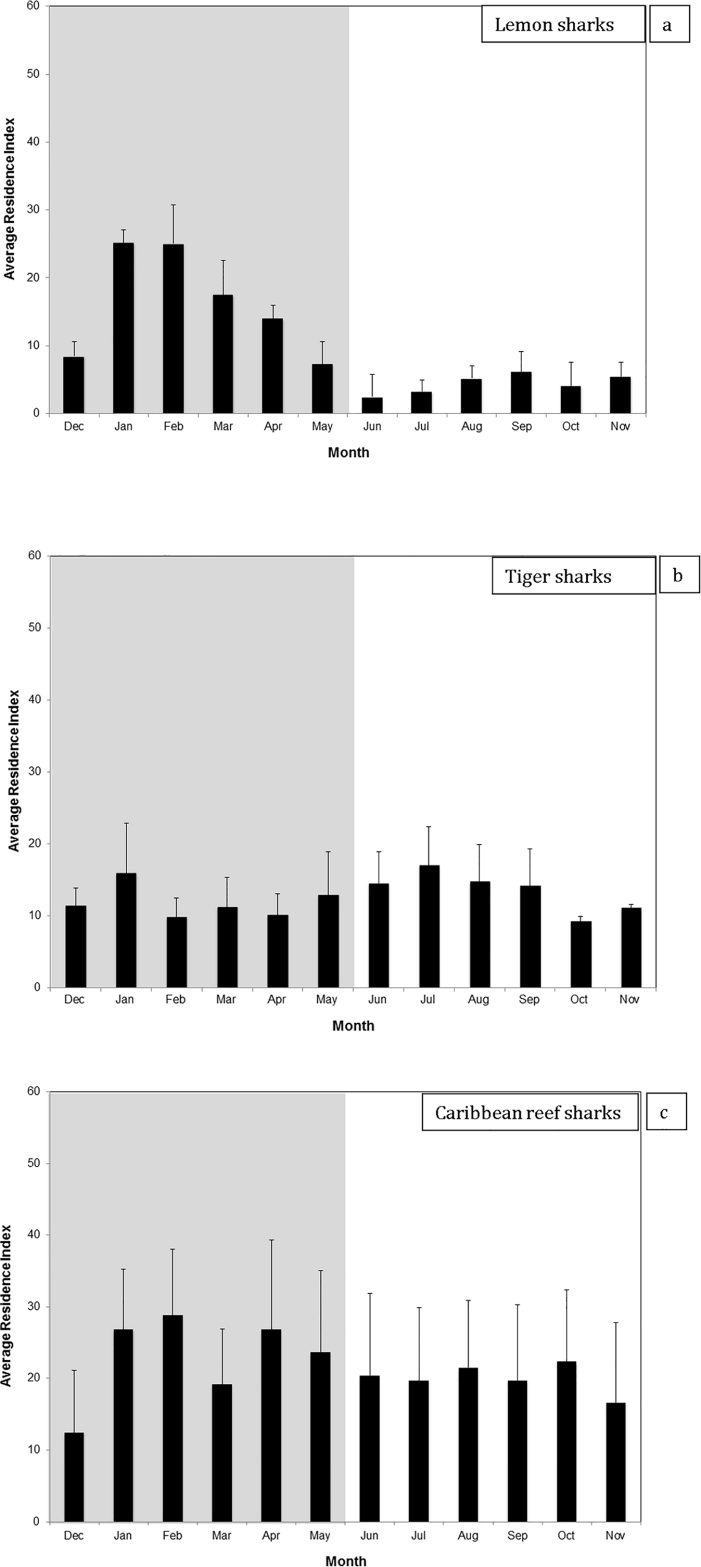
Average Residence Index on a monthly basis (number of days detected at the FSA site as a proportion of the total number of days in the month) between 2007-2011for a) lemon sharks (n = 6); b) tiger sharks (n = 10); and Caribbean reef sharks (n = 2). The gray background represents the grouper spawning season and the white background is non-spawning months. Bars represent mean and standard error.

The degree to which sharks were associated with the FSA site during the grouper spawning season (Dec–May) varied distinctly among species. Lemon sharks demonstrated a marked increase in activity centered in the vicinity of the FSA during grouper spawning months, whereas tiger and Caribbean reef sharks did not exhibit orientation towards the FSA in relation to grouper spawning. There was no significant difference in presence values for lemon sharks at the three discrete spawning areas (*p* = 0.247) indicating that treatment of the MCD and Grammanik Bank as a single FSA for analyses was appropriate. Combining all monthly residence indices for all years for each species yielded a total of 12 monthly average residence indices for each species over the five year span of the study (Fig 5). Residence index was significantly greater during spawning months than non-spawning months (t-test, *p* = 0.008) for lemon sharks, but not for tiger or Caribbean reef sharks (p > 0.05). Additionally, the average number of days per month that lemon sharks were detected at the FSA over the five year span of tracking (4.9 ± 2.3 d) was significantly greater than during the non-spawning season (1.4 ± 0.43 d) (*p* = 0.011). There was no significant difference between FSA residence indices during spawning and non-spawning seasons for tiger sharks (*p* = 0.411). Residence indices of Caribbean reef sharks were not analyzed because only two sharks were tracked.

**Fig 6:**
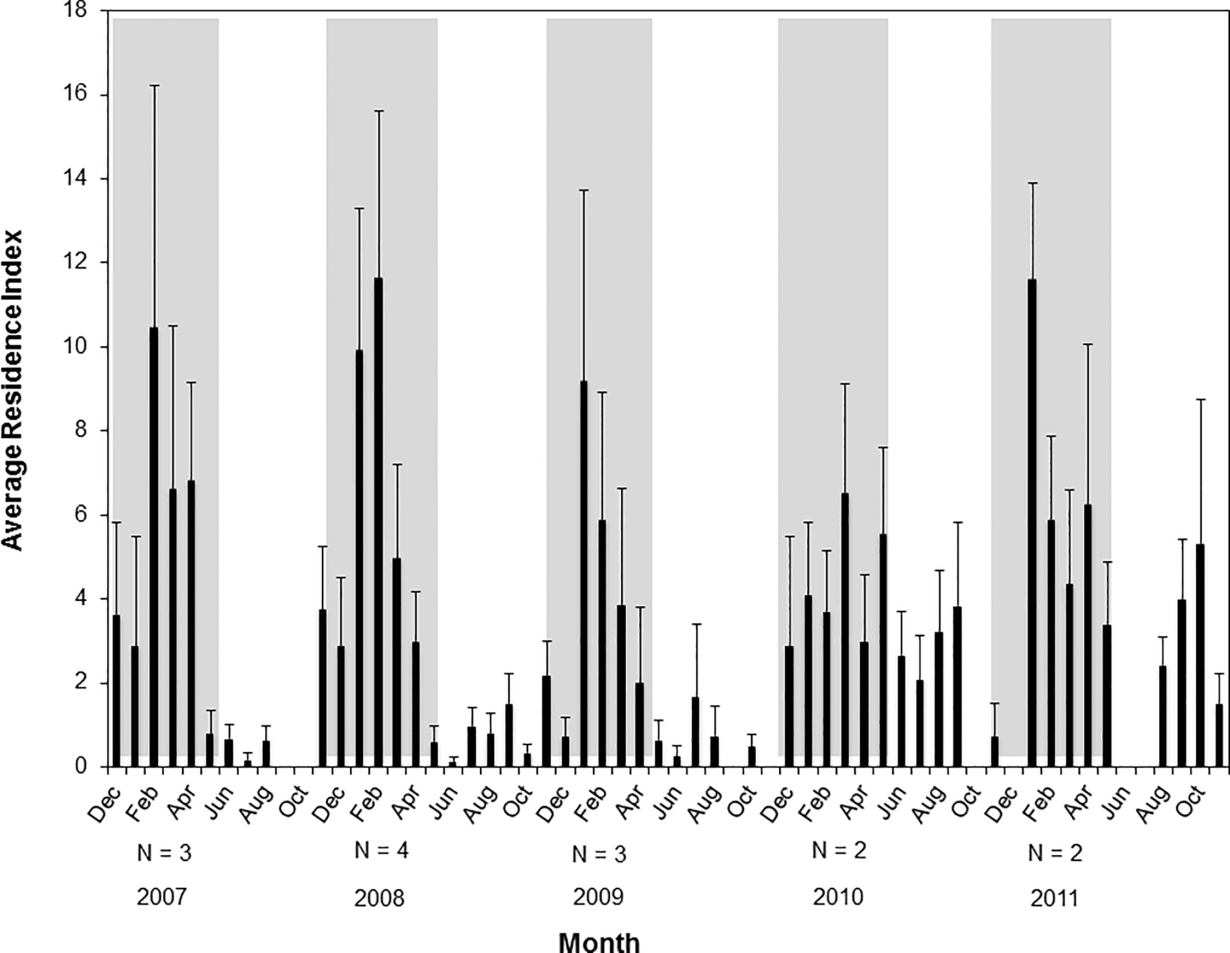
Average Residence Index for lemon sharks at the FSA site during grouper spawning season (gray) and non-spawning season (white) between 2007–2011. Sample size indicates the number of sharks detected each year. Bars represent mean and standard error.

**Fig 7:**
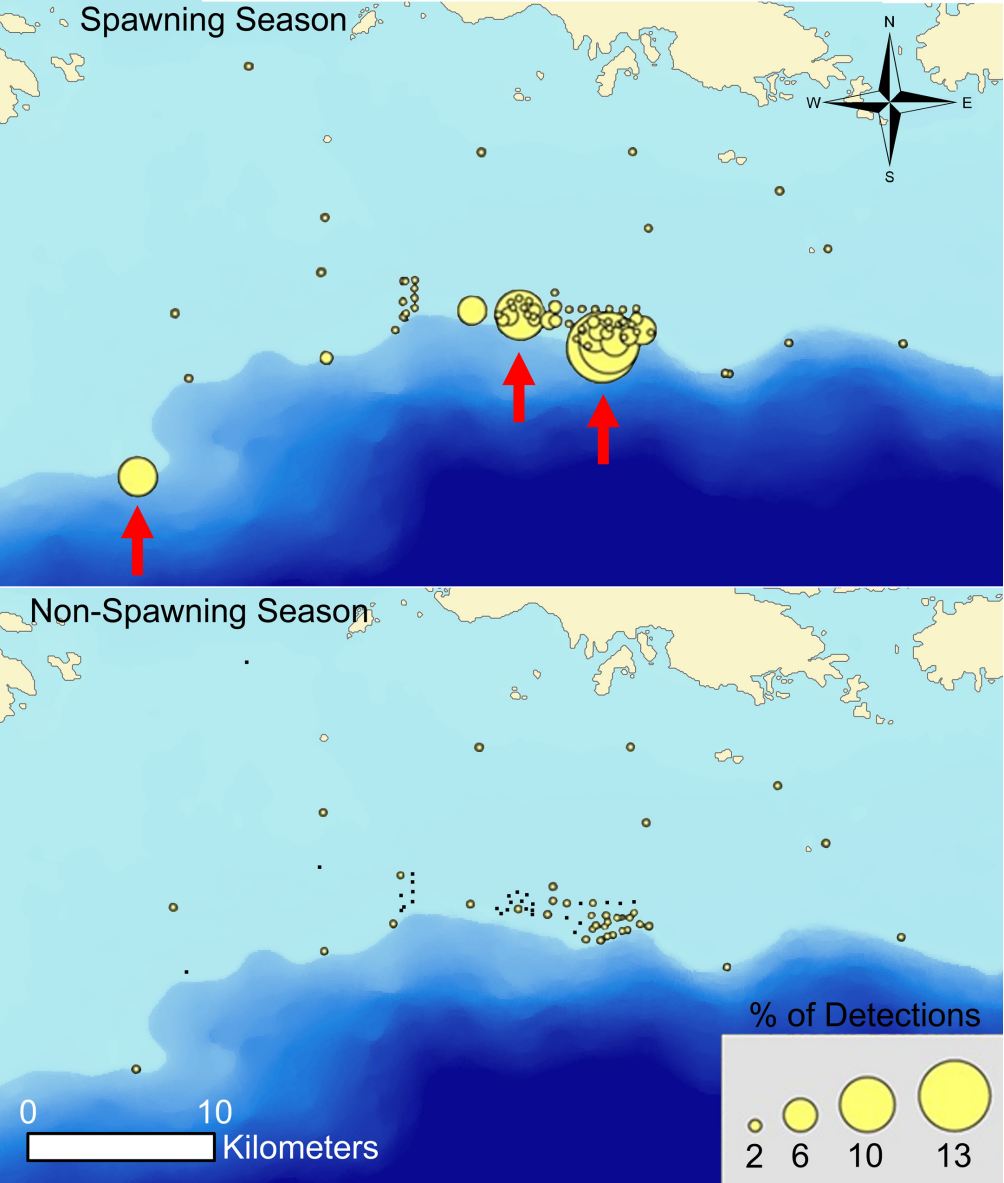
Detections on receivers as proportion of total detections on all receivers for lemon sharks during grouper spawning season and non-spawning season 2007–2011. Red arrows represent Grammanik Bank, Hind Bank MCD and El Seco spawning sites (right to left).

Although lemon sharks were tracked for varying lengths of time and the number of individuals tracked each year was low and consisted of different individuals, the seasonal patterns of orientation to the FSA site during spawning seasons followed by low activity at the FSA in non-spawning seasons were consistent for lemon sharks throughout the five year study. Regardless of the number of lemon sharks tracked or which individuals were tracked, residence indices at the FSA’s were elevated during each spawning season, followed by a significant decrease in residence indices during non-spawning over the course of the study (Fig 6). Because lemon sharks were the only species to show a relationship to the FSA, further examination of movements at the FSA site were limited to this species. The average presence value for lemon sharks at the FSA was higher during grouper spawning season (2.4 ± 0.49 d) than during non-spawning season (1.8 ± 0.57 d), but the difference was not significant (*p* = 0.384). Similarly, the average absence value for lemon sharks at the FSA site during non-spawning (29.5 ± 11.5 d) was more than three times the value recorded during spawning season (8.0 ± 2.1 d), but the difference was not significant (*p* = 0.148). Average residence index at the Hind Bank MCD for lemon sharks (9.2 ± 1.8) was significantly higher than at the Grammanik Bank (2.9 ± 0.87) (*p* = 0.016), suggesting that lemon sharks had a greater affinity for the red hind spawning site. Peak detections of lemon sharks at the FSA occurred in January and February, whereas at the El Seco spawning site 95.2% of detections occurred in March. Partitioning receiver detections as a proportion of total detections into spawning and non-spawning seasons clearly illustrated the difference in behavior of lemon sharks during the two seasons, suggesting that grouper spawning has a very strong influence on pattern of MCE use for this species (Fig 7).

## Discussion

Acoustic telemetry methods used in our study generated tens of thousands of detections for sharks carrying transmitters and provided temporal and spatial records of shark movements within the MCE over a five year span. Similar acoustic telemetry methods have also yielded large amounts of data suitable for evaluating the role of mobile predators on reefs [6,22,32]. Although different numbers of individuals of each species were tracked for varying lengths of time and although our acoustic array covered only a limited portion of MCE habitat on the Puerto Rico-Virgin Islands platform, individuals of each species were tracked in excess of three years, and tracks of nearly four years were obtained for two of the species. The acoustic array allowed nearly uninterrupted tracking of Caribbean reef sharks over an extended period of time when they were within the monitored MCE, but more limited tracking of lemon and tiger sharks. Individuals of all three species spent considerable time outside of the MCE, indicating that other habitats may be equally or even more important than the MCE. Our study enabled collection of sufficient data to clearly demonstrate extensive use either spatially or temporally of the MCE by all three species of shark and also to elucidate distinct patterns of use of the MCE among the three species.

### MCE Use

The three species of sharks showed obvious differences in their use of the MCE as demonstrate by sizes of activity space and their detection histories. Caribbean reef sharks limited their movements to a very restricted portion of the MCE, primarily near the platform drop-off, in proximity to the FSA, with extended periods of time during which they were detected on receivers nearly continuously. The presence of the Caribbean reef sharks near the FSA was most likely due to the capture location of these individuals since neither showed a strong affinity to the FSA during grouper spawning and non-spawning periods. Both presence and absence values for Caribbean reef sharks were an order of magnitude higher than those of either tiger or lemon sharks. Caribbean reef sharks were characterized by presence restricted to a limited area within the MCE for long stretches of time, followed by long periods of absence.

Although small sample size precludes robust conclusions about use of the MCE by Caribbean reef sharks, both individuals tracked behaved similarly and occupied small activity spaces, each roughly 5.5 km^2^ in size despite tracks that exceeded two years. Restricted movements typify species of coral reef-associated sharks, including Caribbean reef sharks, with small home ranges (<100 km^2^) reported in a number of studies [14,33–36]. Caribbean reef sharks tracked in the Bahamas spent a high proportion of their time near a single acoustic receiver [34], and juvenile Caribbean reef sharks were year-round residents at small reef sites in Brazil, displaying a lack of seasonal variation in habitat use [37,38]. There was no evidence of seasonal migration or long-distance movements for the Caribbean reef sharks tracked in our study, however seasonal peaks in abundance during summer months [39] and occasional relatively wide-ranging movements (~50 km) have been reported for this species [16,40]. Movements within a restricted area over a prolonged period of time that typically characterize Caribbean reef sharks suggest intense and chronic ecological interactions on a continual basis in MCEs. Limited movements of these sharks also indicate the potential for a high degree of protection for Caribbean reef sharks in MPAs such as the seasonal area closures associated with FSAs such as the USVI study site [16,38].

There were similarities in spatial and temporal patterns of use of the MCE for lemon and tiger sharks. Both species moved extensively throughout the MCE and were detected on a large majority of receivers within the array. However, lemon sharks exhibited conspicuous seasonal movement patterns with a prolonged presence on the reef and in proximity to the FSA during grouper spawning season followed by long periods of absence during non-spawning. Tiger sharks were the least site attached of the sharks tracked, with little seasonal variation in their movement patterns, and were characterized by relatively short visits to the MCE interspersed with slightly longer absences.

Presence and absence values were similar for tiger and lemon sharks, however; the temporal patterns of their use of the MCE revealed marked differences. For example, all lemon sharks tagged were detected on acoustic receivers nearly 1,000 times and tracked for greater than 100 d, generating an average of approximately nine detections per day, whereas tiger sharks were detected an average of only 2.5 times per day. Additionally, the maximum number of consecutive days that a lemon shark was absent (133 d) from the acoustic array was substantially shorter than the maximum absence recorded for a tiger shark (272 d), and lemon sharks were typically absent for less than one week at a time, compared with higher and much more variable absence values for tiger sharks. Temporal presence/absence patterns of lemon sharks were closely associated with their orientation towards the FSA, typified by prolonged presence during spawning season, and long absences during non-spawning; a trend consistent among individuals. Temporal patterns of MCE use for tiger sharks were highly variable among individuals and punctuated by both short presence and absence values.

A number of the lemon sharks tracked were present within the MCE and at the FSA for extended periods of time during grouper spawning season over consecutive years. Although lemon sharks are capable of relatively long distance movements [41,42], lemon sharks tracked in our study appeared to be fairly site attached to the MCE. It is likely that during their absence from the MCE lemon sharks tagged in our study had moved into shallower reef habitat or MCE habitat in other nearby locations.

Tiger sharks are known to cover great distances, including crossing ocean basins, and even on short time-scales may range over thousands of km [21,32,43–46]. Tiger sharks tracked with satellite transmitters at our USVI study ranged hundreds and thousands of km in relatively short times, and frequented deep water away from the MCE [47]. It is therefore likely that the tiger sharks tracked with acoustic transmitters in our study ranged over a much a larger total area of MCE and to other habitats than monitored by the acoustic array in our study. This interpretation is also supported by the observation that for the tiger sharks tracked in our study seven of 17 individuals were detected a small number of times throughout the five years of our study. Given that tiger shark movements have been linked to prey availability in a variety of ecosystems [48–50], regular visits to MCE by some tiger sharks suggests that at least some tiger sharks are involved in extensive predator-prey relationships in MCEs although these interactions appear to be highly variable among individual sharks. However, other individuals spent much less time within the MCE and the level of ecological interactions with other members of MCEs is likely much lower. Tiger sharks exhibit impressive long-distance movements that result in their habitation of a wide variety of disparate ecosystems that span a very wide range of energy densities, ranging from site attached movements on tropical coral reefs to vast, long-term movements within the oceanic province [32,46]. As such, tiger sharks are likely involved in an extremely wide range of ecological interactions with a broad spectrum of organisms within what for sharks may be an unparalleled diversity of ecosystems that they inhabit. Compared to Caribbean reef and lemon sharks, tiger sharks are a more transient species through the MCE, with movements influenced by factors such as season, age, sex and substrate [21,44–46,51]. All of these factors contribute to a multifaceted and complex series of interactions for tiger sharks within the MCE and also various levels of connectivity between the MCE and other ecosystems [6,52].

### Association with the FSA

The three species of sharks oriented to the FSA differently, with only lemon sharks showing clear patterns of movement related to the FSA. Although Caribbean reef sharks are piscivorous [53,54], they exhibited no orientation to the FSA. Similar indifference towards predictable and frequent prey has been reported for Caribbean reef sharks in the Bahamas [36]. Despite long-term daily provisioning at a dive site, Caribbean reef sharks showed no changes in space use and movement, nor did they increase time spent at the feeding site.

Tiger sharks also demonstrated a lack of orientation to the FSA during the grouper spawning season. Although tiger sharks have been reported to remain in specific locations for short periods of time (days to weeks) in association with seasonally abundant prey [21,32,44–46], this species is also characterized by wide-ranging movements [43–47] and their wide dietary breadth [48–50,55,56]. The lack of orientation of tiger sharks to the FSA despite the presence of a large amount of potential prey may be related to a number of factors, including their extremely diverse diet, large size of the sharks tracked, and their wide-ranging movements. The tiger sharks monitored in this study were relatively large, with an average size of 303 cm and larger tiger sharks have been found to rely less on teleost prey and consume more crustaceans, elasmobranchs, and sea turtles [50,55]. The seasonal abundance of teleost prey at the FSA is apparently not sufficient to alter tiger shark behaviors to the degree to which they focus on aggregations of spawning grouper and other teleost fishes.

Lemon sharks exhibited a marked orientation to the FSA during grouper spawning season, a pattern that occurred consistently during each of the five years of the study. The chronic nature of this association is evidenced by increased presence of individual lemon sharks at the FSA during each grouper spawning season for as long as four consecutive spawning seasons. Additionally, although only two lemon sharks were detected at the El Seco site, over 95% of their detections were recorded during peak spawning of tiger grouper, the predominant grouper species spawning at that location [30]. Tiger grouper spawning peaks several months after peak spawning at the Hind Bank MCD and Grammanik Bank (R. Nemeth, pers. obs.), further suggesting that lemon sharks specifically orient to spawning events. While it is not clear if lemon sharks feed on spawning grouper or on other teleosts such as plankivores that are attracted to grouper eggs released at spawning sites, the diet of lemon sharks consists of a high proportion of teleosts [57]. Seasonal periodicity of lemon sharks at spawning sites is likely related to the predictable presence of a large amount of potential prey, as has been reported for a number of predators in relation to spawning sites or other predictable influxes of prey [58–61]. One such predator is the sickle fin lemon shark (*Negaprion acutidens*), which are thought to have learned to return to a particular area where food is spatially and temporally predictable [62]. The observation that lemon shark movements are heavily influenced by grouper spawning activities may be beneficial to lemon sharks that are seasonal residents within areas that are closed to fishing. MPAs likely provide minimal protection from fishing pressure for more highly mobile species such as tiger sharks, underscoring the challenges of conservation of sharks that undergo large-scale movements [22,51,63,64].

## Conclusions

The MCE on the Puerto Rico-Virgin Islands platform was used extensively, year round by all three species of sharks studied. However, each species exhibited different patterns of use of the MCE and orientation towards spawning sites. Caribbean reef sharks were detected throughout the year within a small area exclusively within the FSA site. They exhibited long residence times but occasionally were absent from the array for over a year before returning. Tiger sharks used the majority of the MCE habitat monitored and were typically detected within the acoustic array over short number of consecutive days throughout the year, interspersed by short absences. Lemon sharks also used nearly the entire MCE habitat monitored by the acoustic array, but exhibited a strong association with the FSA and El Seco site during the grouper spawning season. Both the frequency and duration of visits to the MCE by all three species suggest that these species are regularly present on the reef and are likely involved in a wide array of ecological interactions within the MCE, but variable in nature.

The high degree of orientation of lemon sharks to spawning sites demonstrates the influence of prey on movements of predators and illustrates the potential of sharks to interact with fishes at spawning sites. The strong association of lemon sharks with multiple spawning sites emphasizes the potential importance of spawning events in shaping ecosystem dynamics of MCEs and also illustrates the potential for predation on commercially important fishes, such as groupers, to influence their populations and to warrant consideration in efforts to manage these stocks.
